# Angstrom-Resolved Metal-Organic Framework-Liquid Interfaces

**DOI:** 10.1038/s41598-017-11479-4

**Published:** 2017-09-11

**Authors:** Stefano Chiodini, Daniel Reinares-Fisac, Francisco M. Espinosa, Enrique Gutiérrez-Puebla, Angeles Monge, Felipe Gándara, Ricardo Garcia

**Affiliations:** Materials Science Factory, Instituto de Ciencia de Materiales de Madrid, CSIC, c/ Sor Juana Inés de la Cruz 3, 28049 Madrid, Spain

## Abstract

Metal-organic frameworks (MOFs) are a class of crystalline materials with a variety of applications in gas storage, catalysis, drug delivery or light harvesting. The optimization of those applications requires the characterization of MOF structure in the relevant environment. Dynamic force microscopy has been applied to follow dynamic processes of metal-organic-framework material. We provide images with spatial and time resolutions, respectively, of angstrom and seconds that show that Ce-RPF-8 surfaces immersed in water and glycerol experience a surface reconstruction process that is characterized by the diffusion of the molecular species along the step edges of the open terraces. The rate of the surface reconstruction process depends on the liquid. In water it happens spontaneously while in glycerol is triggered by applying an external force.

## Introduction

Atomic, molecular and nanoscale resolution images of surfaces and interfaces in technologically relevant conditions are critical to predict and understand the behavior of functional materials and devices^1–3^. Metal-Organic Frameworks (MOFs)^4^ are a class of materials that are drawing considerable attention due to their multiple applications in fields such as gas storage, catalysis, drug delivery, optics, or sensing, among others^5–8^. MOFs are crystalline solids built by the joining of metal ions or cluster through organic linkers, to assembly periodic structures with desired topologies. Recent developments emphasize the formation of MOFs able to display a combination of heterogeneity and order. Thus, multi-variate (MTV) MOFs, which are built by the combination of multiple linkers with different functionalities, have shown enhanced gas sorption capabilities^9^, multi-metal or solid solution MOFs have demonstrated controllable catalytic activity^10^, or defect-engineered MOFs are currently emerging as complex materials with tunable sorption (and related) properties^11^. The high crystallinity of MOFs has traditionally allowed their structural characterization with the use of X-ray diffraction techniques^12^, and current efforts are being made to implement the use of high resolution electron microscopy techniques for the study of this class of materials^13^. However, the increase in the degree of complexity and the variety of MOFs applications are demanding the implementation and/or development of novel characterization techniques to enable the understanding of the structure-property relationship in their native functional environment. Those methods should have atomic resolution and enable the characterization of dynamic processes. The atomic force microscope (AFM) has demonstrated its capability to image with high resolution a variety of surfaces and materials in different environments from vacuum^14–16^, to different gas environments^17, 18^, to liquid^19–25^.

In this letter we present a dynamic force microscopy method to follow with molecular resolution the evolution of a MOF-liquid interface. The method resolves the positions of the central metal atom and the organic linkers. It has a lateral resolution of 0.3 nm. We provide evidence of the removal of molecular clusters involving 11 atoms along the step edges. The images support a step-like process that involves the removal of half of the Ce cations of the unit cell. This process introduces significant changes on the surface of the material. The surface reconstruction rate depends on the liquid. In water the surface reconstruction happens spontaneously while in a viscous liquid such as glycerol, the surface reconstruction process is induced by the force applied by the AFM probe. We demonstrate the capability of amplitude modulation AFM to characterize the interaction of MOF surfaces in technological relevant environments by providing images of the surfaces and etching processes in the native environment with angstrom resolution.

**Figure 1:**
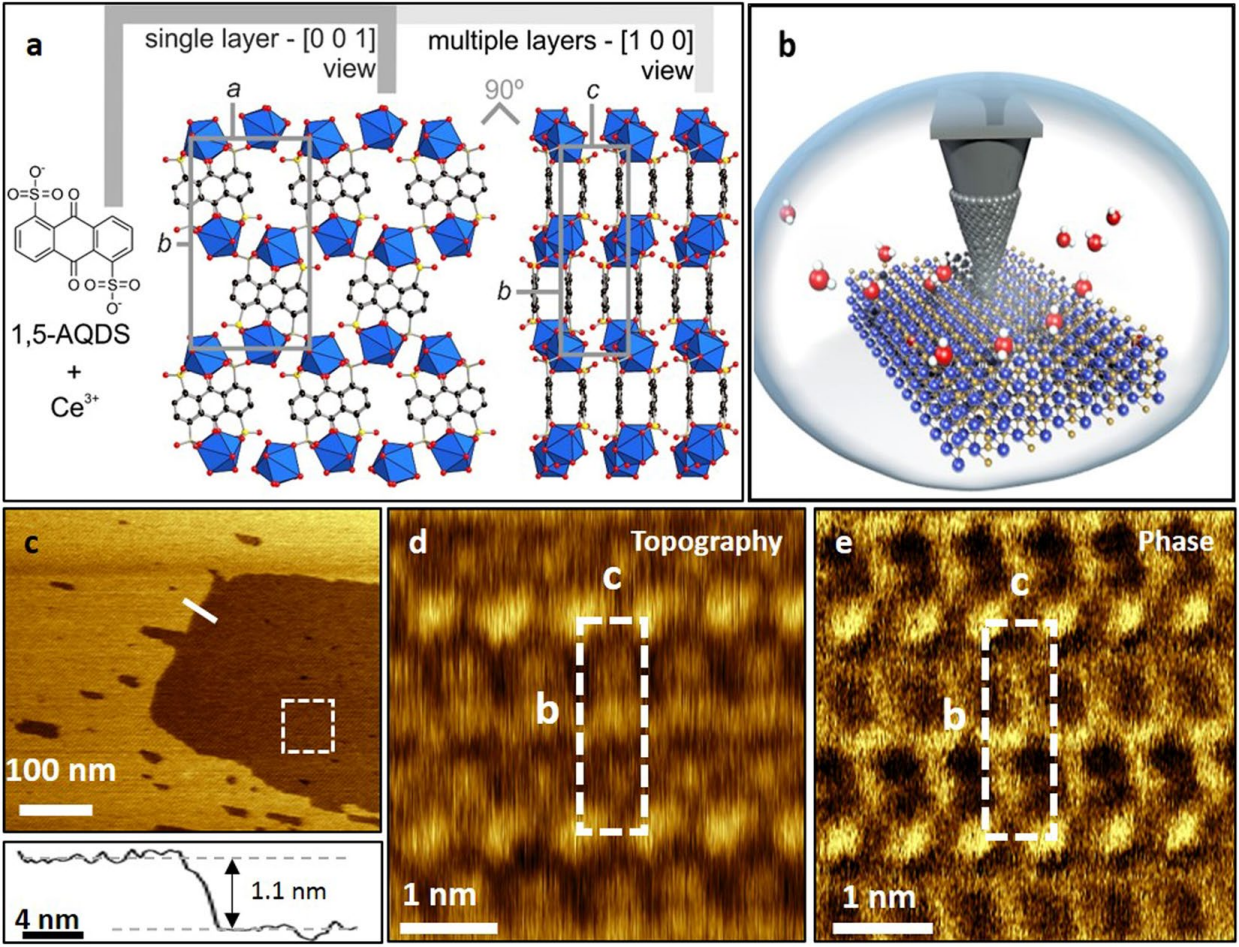
Structure of the MOF and AFM images. (**a**) The structure of Ce-RPF-8 consists of double layers disposed perpendicular to the *ab* plane. Top and side view of the layers are depicted in the figure, showing unit cell dimensions. Cerium atoms are represented as blue polyhedra, carbon, sulfur and oxygen atoms are black, yellow and red balls, respectively. (**b**) Schematic illustration of the AFM experimental set-up in liquid. (**c**) AFM topography image of a region of a Ce-RPF-8 *bc* plane. The image has been obtained in glycerol. (**d**) Molecular resolution image of the region marked in (**c**) (topography). (**e**) Phase contrast image of the region marked in (**c**). Imaging parameters for panels d,e: *A*_0_ = 0.5 nm, *A*_sp_ = 0.14 nm, fast axis scanning frequency = 20 Hz.

For this study, we have selected a MOF belonging to the family of RPF-8 (rare earth polymeric framework-8) materials, previously reported by us^26^. This MOF family is made of the combination of rare-earth cations, and a radical organic linker (anthraquinone-1,5-disulfonic acid, AQDS), conferring the materials with high charge mobility. In particular, we have prepared the cerium version of this MOF type. Figure 1a shows a scheme of the structure of Ce-RPF-8, as determined by single-crystal X-ray diffraction (see Supplementary Information for details of crystallographic analysis). This compound crystallizes in the orthorhombic *P*2_1_2_1_2 space group, with lattice parameter *a* = 1.1472 nm, *b* = 2.0947 nm, *c* = 0.7087 nm. The structure consists of cerium cations coordinated to the sulfonate group of the linkers, as well as to the linker quinonic oxygen atoms. Three water molecules complete the coordination sphere of the cerium atoms in the crystal. The linkers are stacked in a face-to-face disposition along the *c* axis, forming double layers that are parallel to the *ab* plane. Intra- and inter-layer distances are 0.3251 nm and 0.3836 nm, according to the X-ray diffraction data (Fig. 1a). Figure 1b shows a scheme of the AFM tip-MOF interface in liquid. In Fig. 1c we show an amplitude modulation AFM topographic image of the Ce-RPF-8 surface immersed in glycerol. The image shows two terraces separated by a step of 1.1 nm in height (cross-section). Figure 1d,e show, respectively, the topography and the phase contrast image of a small region of the surface. The AFM images show a periodic structure which lattice values of 2.1 nm (*b* unit vector) and 0.7 nm (*c* unit vector). In addition, the step size (Fig. 1c) matches the value of the *a* lattice parameter (Fig. 1a). Those values and the overall structural symmetries match the X-ray data reported for the (100) face of the MOF. The position of the Ce atoms as well as the organic linkers are observed in both the topography and phase contrast images. It has been established that phase shift channel provides higher contrast on heterogeneous materials than the height channel^27^. This is also reflected here (Fig. 1e). The phase contrast AFM image shows features associated with the organic linkers that have angstrom spatial resolution (0.3 nm).

The AFM images show an asymmetry in the contrast given by the position of the Ce atoms in the *bc* lattice plane. The intermediate Ce atoms give a weaker topography signal. This observation is in agreement with the X-ray data (Fig. 1a) that shows the position of the intermediate Ce cations shifted downwards about 0.1 nm from the line defined by the corners of the *ab* lattice plane. We also observe that the position of the organic linkers in the phase contrast image is slightly offset with respect to the X-ray data (Fig. 1a). For surfaces containing a variety of atomic species, the interpretation of AFM topographic and phase contrast images in terms of individual atomic features requires the use of atomistic simulations that include the effects associated with the charge of the surface atoms and the atom distribution on the tip apex^28^. The use of those simulations is beyond the scope of this contribution.

**Figure 2:**
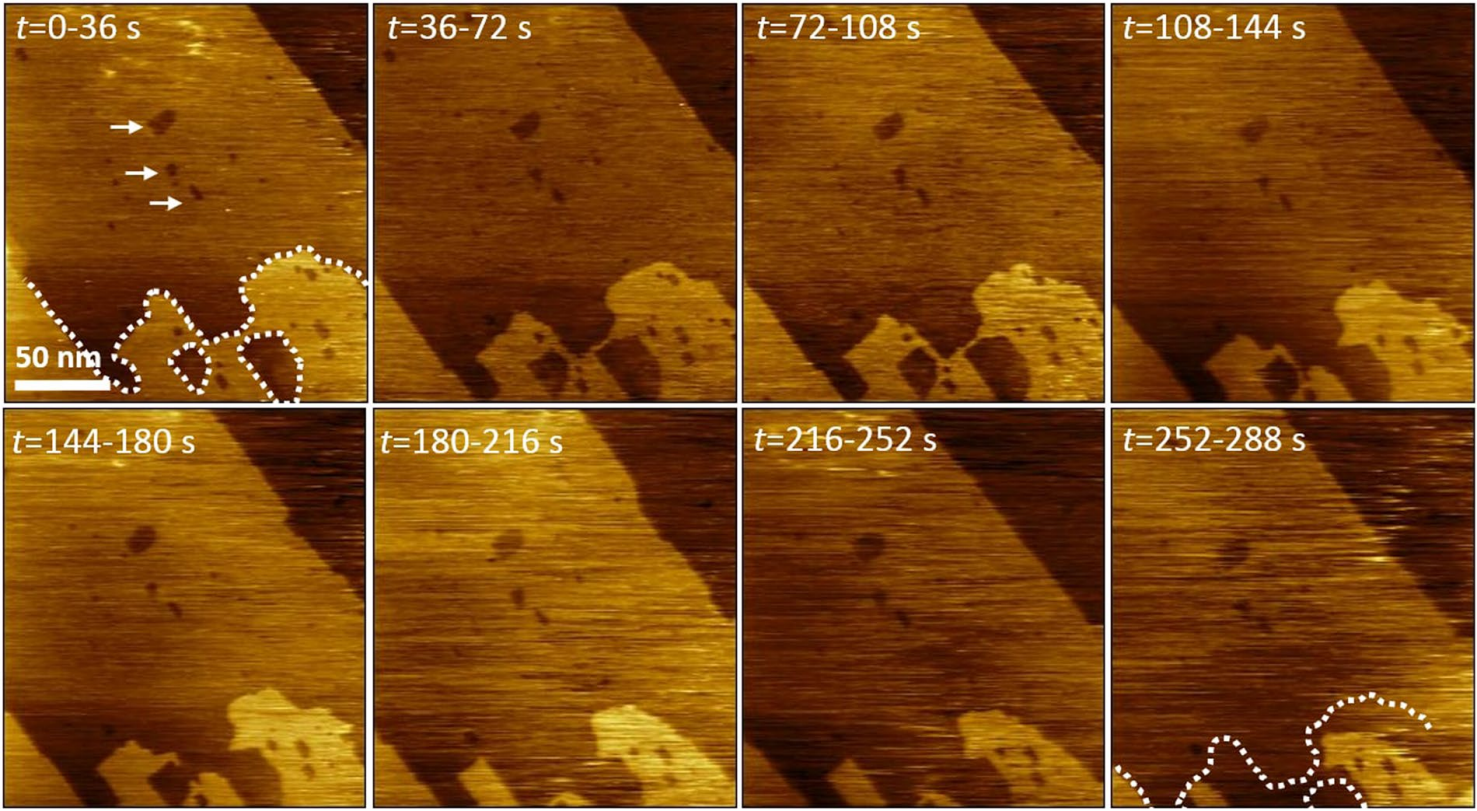
Sequence of AFM images showing MOF etching phenomena in water. Sequence of amplitude modulation AFM images (topography) obtained with the MOF immersed in water. The contour of the top terrace at the beginning of the observation is marked. Imaging parameters: *A*_0_ = 5.2 nm, *A*_sp_ = 1.8 nm, fast axis scanning frequency = 7 Hz.

Figure 2 shows a sequence of images of the Ce-RPF-8 sample immersed in distilled water (pH = 5.5). The sequence covers a time span of about 5 min. The associated video is included in the Supplementary Information (Supplementary Video S1). The initial frame shows two molecular steps that separate three terraces. The top and central terrace show several nanometer-scale vacancy islands. We have marked the edges of the initial top terrace to track the visualization of the surface changes. The frontline of the top terrace recedes with the observation time. The large vacancy islands situated on the lower bottom of the top terrace become larger and eventually coalesce. The process seems to happen by thinning the bridge separating the intermediate terrace and the vacancy islands. On the other hand, the three smaller vacancy islands situated in the middle of the intermediate terrace remain practically unchanged. Those islands are marked by arrows in the initial frame (*t* = 0–36 s). This observation indicates the heterogeneous character of the surface modification process. The surface modification process of the MOF surface in water happens at a rate that is too fast for acquiring images with molecular resolution.

It is important to note that the bulk Ce-RPF-8 crystals are stable in water over a 24 hour period. To study the existence of a MOF dissolution process, a batch sample of Ce-RPF-8 crystals was immersed in water for 24 hours. Optical microscopy images of the MOFs did not show any significant differences before and after a 24 hour immersion in water (Figure S1). In addition, we have performed Powder X-ray Diffraction (PXRD) experiments before and after immersion in water. Those patterns (Figure S2) are confirming that the bulk MOF crystal is stable in water.

**Figure 3:**
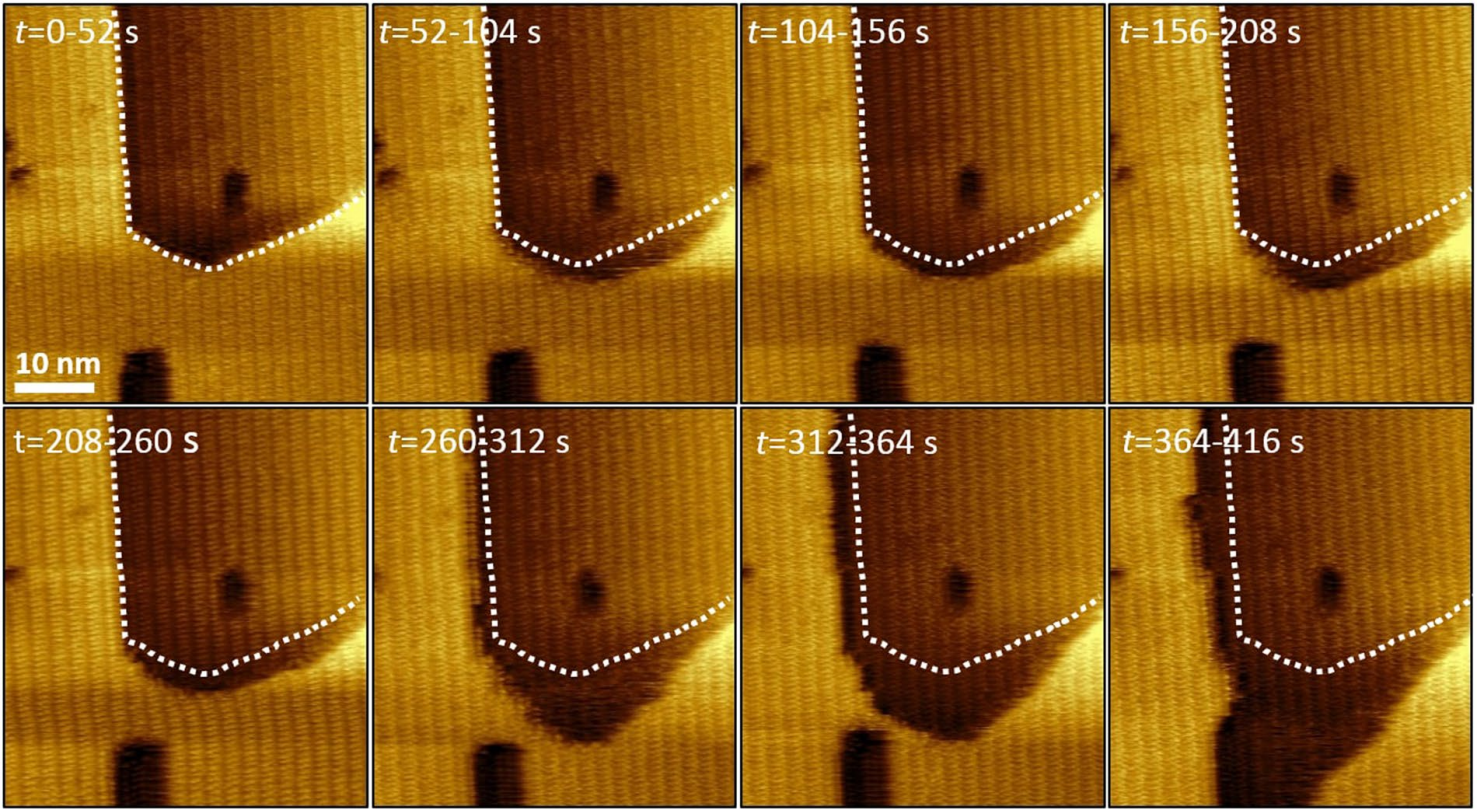
Sequence of AFM images showing MOF etching phenomena in glycerol. Sequence of amplitude modulation AFM images (topography) obtained with the MOF immersed in glycerol. The contour of the top terrace at the beginning of the observation is marked in all the frames. Imaging parameters: *A*_0_ = 0.44 nm, *A*_sp_ = 0.15 nm, fast axis scanning frequency = 20 Hz.

To follow the modification of Ce-RPF-8 surfaces with angstrom resolution we have replaced water by glycerol. Glycerol is a viscous liquid. Figure 3 shows a sequence of AFM images obtained by immersing the MOF crystal in glycerol. The associated video is included in the Supplementary Information (Supplementary Video S2). The frames show two MOF terraces separated by a molecular step. In all the frames, the molecular ordering along the *b* axis of the crystal (2.1 nm) is visible on both terraces. We have marked the edges of the initial top terrace to track with molecular resolution the surface changes. The sequence of frames shows that the terrace width decreases by about 2 nm per minute. The surface reconstruction process is characterized by the changes in the profile of the step edges. The removal of the atoms from the edge of the upper terrace decreases the distance to the rectangular island observed in the lower left corner of the image. Eventually, the rectangular island merges with the lower terrace, which produces a sudden decrease of the upper terrace size. The 16 nm^2^ vacancy island observed in the middle of the central terrace is used as a reference point to follow the surface modification process. The size of the island is quite stable over the observation time. This indicates that, at least initially, the atoms remain on the terrace without diffusing into the solvent.

**Figure 4:**
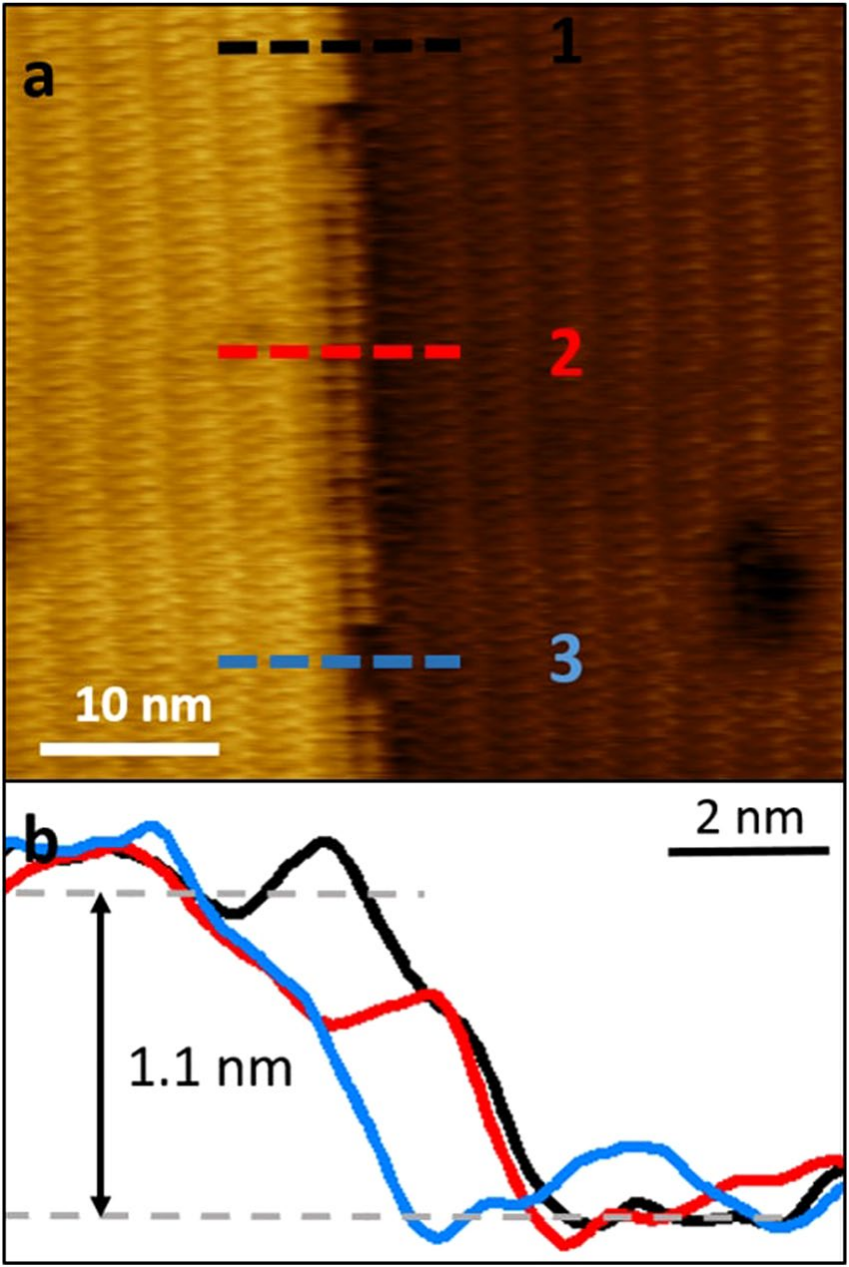
AFM image showing half MOF layer removal in glycerol. Amplitude modulation AFM image of a step edge a Ce-RPF-8 sample. Three structures of the edge are observed. A perfect edge; a section of the edge with a half layer and the total removal of the unit cell. (**b**) Height cross-section along the lines marked in (**a**).

We have also obtained images that illustrate the removal of half of the Ce cations along the *a* axis. Figure 4a shows an AFM image of a step edge with three different sections. One section includes the full width of the step, another one shows the removal of the top Ce cations of the edge, and in the other one the removal of all the cations of the unit cell along the *b* axis. The profiles (Fig. 4b) shows a height change of 1.1 nm between top and bottom terrace in agreement with the *a* lattice parameter. The profile along the line marked as 2 shows two steps separated by 0.5 and 1.1 nm from the top terrace. This profile indicates that the step edge modification happens in a step-like manner with the removal of half of the Ce cations along the *b* axis. The profile 3 shows a section of the edge without the whole unit cell.

**Figure 5:**
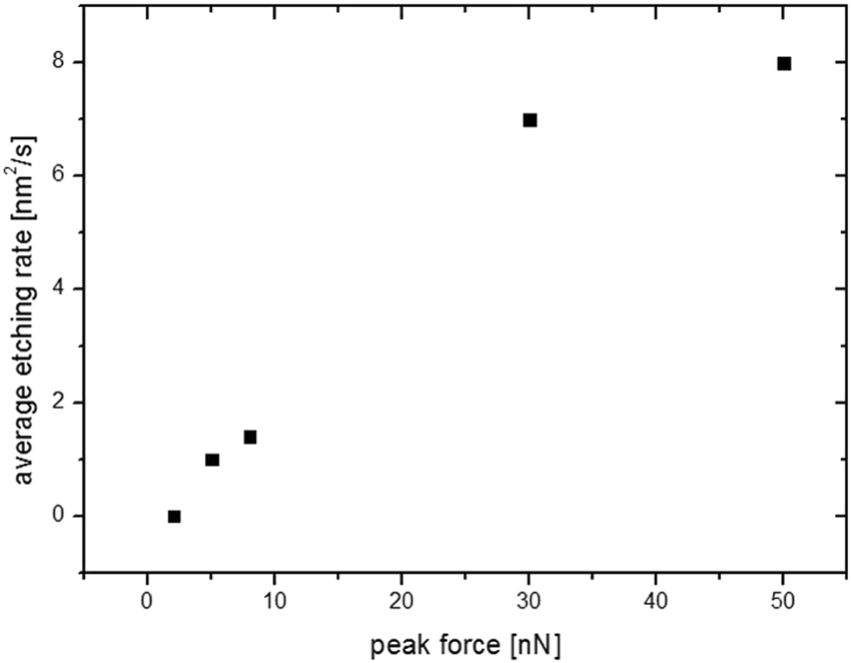
Average etching rate vs peak force plot. Terrace etching rate as a function of the applied force for a Ce-RPF-8 sample immersed in glycerol.

We have measured the average change of the surface area of the top terrace as a function of the applied peak force^29^ (see Supplementary Information). The data in Fig. 5 shows that the etching rate increases with the applied force or the stress until a saturation value is found. At an applied force of 10 nN, or its equivalent in stress (1 GPa), a 2 nm^2^ s^−1^ surface rate change is observed. More importantly for forces below 2 nN no surface changes are observed.

The observations in water and glycerol indicate that the surface reconstruction process starts with the diffusion of the MOF atoms along the step edges of the open terraces to other regions of the crystal that act as a sink for the atoms.

In summary we report angstrom-resolved images of the surface changes of a MOF surface immersed in liquid. Our findings show that Ce-RPF-8 surfaces immersed in water and glycerol experience a surface reconstruction process that is characterized by the diffusion of the molecular species along the step edges of the open terraces. The step modification happens in a step-like fashion by removing half of the cations of the unit cells at the step edges. The rate of the surface reconstruction process is liquid dependent. In water it happens spontaneously while in glycerol is triggered by applying an external force. These results underline the capability of amplitude modulation AFM to follow the dynamics of the surface reconstruction processes with atomic and/or molecular resolution of MOF surfaces in native environments. The data will enable to optimize the performance of MOF applications.

## Methods

The imaging has been performed in amplitude modulation AFM^30^ by photothermally exciting the microcantilever at its 1^st^ resonance. We have used a range of free *A*_0_ and set point amplitudes *A*_sp_. In particular, the atomic resolution images have been obtained by using very small set-point amplitudes ≈0.2 nm and a relatively fast raster frequency (~20 Hz). Information about the AFM imaging conditions are detailed in the Supplementary Information.

## Electronic supplementary material

Supplementary Information

Supplementary Video 1

Supplementary Video 2
