# Supplementary material for: Angstrom-Resolved Metal-Organic Framework-Liquid Interfaces
Source: Sci Rep. 2017 Sep 11;7:11088. doi: 10.1038/s41598-017-11479-4 (PMC5593909; doi:10.1038/s41598-017-11479-4)
Supplement: Supplementary file 1 — Supplementary Information [file 41598_2017_11479_MOESM1_ESM.pdf]

Supplementary Information

Angstrom-resolved Metal-Organic Framework-liquid interfaces

Stefano Chiodini, Daniel Reinares-Fisac, Francisco M. Espinosa, Enrique Gutiérrez-Puebla,

Angeles Monge, Felipe Gándara, Ricardo Garcia

Synthesis and X-ray structural characterization of Ce-RPF-8

60 mg of $Ce(NO_3)_3 \cdot H_2O$ and 120 mg of 9,10-dioxo-9,10-dihydroanthracene-1,5-disulfonic acid, disodium salt (1,5-$AQDSNa_2$) were placed in a Teflon vessel. A solvent mixture composed of 3 mL of water, 8 mL of n-butanol, and 0.1 mL of formic acid were added to the vessel. The mixture was stirred at room temperature for 5 minutes, and then the vessel was introduced in a stainless steel autoclave. The autoclave was heated in an oven at 170 °C for 18 hours. After cooling to room temperature, the solid was filtered and then washed with water (20 mL) and acetone (50 mL). Black, prismatic crystals of Ce-RPF-8 were obtained. One of them was selected under an optical microscope to carry out a single crystal X-ray diffraction experiment. The crystal was mounted with oil on a cryoloop, and placed on a Bruker four circle kappa-diffractometer equipped with a Cu INCOATEC microfocused source, operated at 30 W power (45 kV, 0.60 mA) to generate Cu Kα radiation (λ = 1.54178 Å), and a Bruker VANTEC 500 area detector (microgap technology). The diffraction data were collected exploring over a hemisphere of the reciprocal space in a combination of φ and ω scans to reach a resolution of 0.85 Å, using a Bruker APEX2 software suite. Unit cell dimensions were determined for least-squares fit of reflections with $I > 5\sigma$. Space group determination was carried out using XPREP. The structures were solved by direct methods. The final cycles of refinement were carried out by full-matrix least-squares analyses with anisotropic thermal parameters of all non-hydrogen atoms. The hydrogen atoms were fixed at their calculated positions using distances and angle constraints. All calculations were performed using APEX2 software for data collection and SHELXTL2 and OLEX2-1.22 to resolve and refine the structure. Crystallographic data for $C_{14}H_{12}CeO_{11}S_2$, Ce-RPF-8: Mw: 560.48g mol-1. Orthorhombic, $P2_12_12$, a = 11.4722(6) Å, b = 20.9474(11) Å, c = 7.0874(4) Å, V = 1703.19(16) $Å^3$, D_{calc} = 2.186 g cm^{-3}. F(000) = 1096.0, GOF on F^2 = 1.196, R_1 ($I > 2\sigma$) = 0.0314, wR_2 (all data) = 0.1240.

The purity of the bulk sample was monitored by comparison of the experimental powder X-ray diffraction pattern with the one calculated from the single crystal X-ray analysis.

Sample preparation

Polished silicon samples were cleaned in three cycles with a solution of 1:1:5 in volume of ammonium hydroxide (30%), hydrogen peroxide(30%), and pure water by ultrasonic treatment for 10 minutes each. After that, an additional cycle of 5 minutes in deionized water was done. Subsequently, the silicon surface was spin coated with a mixture of polydimethylsiloxane curing agent (PDMS), PDMS elastomer base (Sylgard 184, Sigma Aldrich) and hexane (Scharlau, Scharlab, S.L.), with a proportion of 1:10:1000 (by weight) at 5000 rpm for 60 s. Ce-RPF-8 crystals were deposited on the PDMS surface and cured on a hot plate at 90^{0}C for 40 min. Finally, Ce-RPF-8 crystals which were not properly attached, were removed by ultrasonic treatment of five seconds in deionized water and the sample was dried with N_2.

AFM imaging

The AFM images were all obtained using a commercial Cypher microscope (Asylum Research, Oxford Instruments). We have placed on top of the sample a droplet of ultra-pure water (18.2 MΩ, pH=5.5) or glycerol (99%, Sigma-Aldrich) waiting 30 minutes before scanning. This allows the cantilever-droplet-sample system to thermal equilibrate before acquiring any image, therefore minimizing the thermal drift.

A photo-thermal excitation of the cantilevers was used consisting of a blue laser spot (wavelength 405 nm) focused onto the base of the cantilever. The used (Filter Cube) gain factors to produce the driving excitation of the cantilever were 0.3 x for Arrow UHFAuD (glycerol experiments), and 0.1 x for RC800PSA (water experiments).

Amplitude modulation AFM operational parameters

Each cantilever spring constant was calibrated on mica, while the resonance frequency and the Q factor were obtained, before the experiments, by fitting the thermal response of the cantilever away from the surface, in the corresponding liquid (water or glycerol, see below).

The images performed in water (Fig. 2 and Supplementary Video S1) were obtained with an RC800PSA (Olympus, Asylum Research, Oxford Instruments) cantilever, $k \approx 0.5$ N m^{-1}, $f \approx 25$ kHz, $Q \approx 3$ (both in water).

All the topographies obtained in glycerol (Fig. 1c, d, e, Fig. 3, 4 and Supplementary Video S2), instead, were achieved with ARROW UHFAuD cantilevers, (NanoWorld, radius < 10 nm), with the following characteristics (in glycerol) for the first resonance: $k \approx 4$ N m^{-1}, $f \approx 100$ kHz, $Q \approx 0.3$. The processing of the AFM images was done by Gwyddion[S1].

Additional AFM imaging parameters

Figure 1c. Set-Point amplitude (SP) = 0.77 nm, free amplitude (A0) = 0.84 nm, scan resolution = 512 px, scan rate = 2 Hz. Image processing consisted of a flattening by means of the Gwyddion option “level rows using intersections with given lines” with the lines all taken on the top terrace. Moreover, a tilting in the x direction was operated.

The height profile shown in the bottom left part of Fig. 1, was obtained averaging five single height profiles taken in different zones of the step of Fig. 1c.

Figure 1d. SP = 0.15 nm, A0 = 0.5 nm, scan resolution = 1024 px, scan rate = 20 Hz.

Image processing consisted of a flattening plus a 2nd order polynomial alignment of rows. The raw image showed unit lattice vectors equal to 2.5 nm x 0.9 nm, therefore it was drift corrected in order to obtain the proper x-ray measurements, 2.1 nm x 0.7 nm. The Gwyddion options for this step were: “Distortion→Affine”.

Figure 1e. SP = 0.14 nm, A0 = 0.5 nm, scan resolution = 1024 px, scan rate = 20 Hz. The image was not processed by Gwyddion, the only difference between the raw image and Fig. 1e is a proper stretching of the color range in order to enhance the contrast. The raw image showed unit lattice vectors equal to 2.5 nm x 0.8 nm, therefore it was drift corrected to obtain the x-ray measurements, 2.1 nm x 0.7 nm.

Figure 2. SP = 1.8 nm, A0 = 5.2 nm, scan resolution = 256 px, scan rate = 7 Hz. Image processing consisted of a flattening plus a 2nd order polynomial alignment of rows.

Figure 3. SP = 0.15 nm, A0 = 0.44 nm, scan resolution = 1024 px, scan rate = 20 Hz. Image processing consisted of a flattening plus a 2nd order polynomial alignment of rows.

Figure 4. SP = 0.18 nm, A0 = 0.45 nm, scan resolution = 1024 px, scan rate = 20 Hz. Image processing consisted of a flattening plus a 2nd order polynomial alignment of rows.

Figure 5. the average etching rate corresponding to a specific peak force was calculated by Gwyddion with a standard threshold procedure which calculate the percentage of the image covered by the layer under investigation. This procedure was done for each couple of consecutive image, therefore obtaining, for each couple, an etching rate. Finally, the average etching rate was calculated. On the other hand, the peak force was obtained by dForce[S2]. Simulation parameters were: the imaging values corresponding to the image under investigation, plus an Hamaker constant approximated to zero since the experiments were performed in (polar) liquids, a MOF Young modulus of 30 GPa and a tip radius of 5 nm. The short-range interaction was simulated with a DMT model.

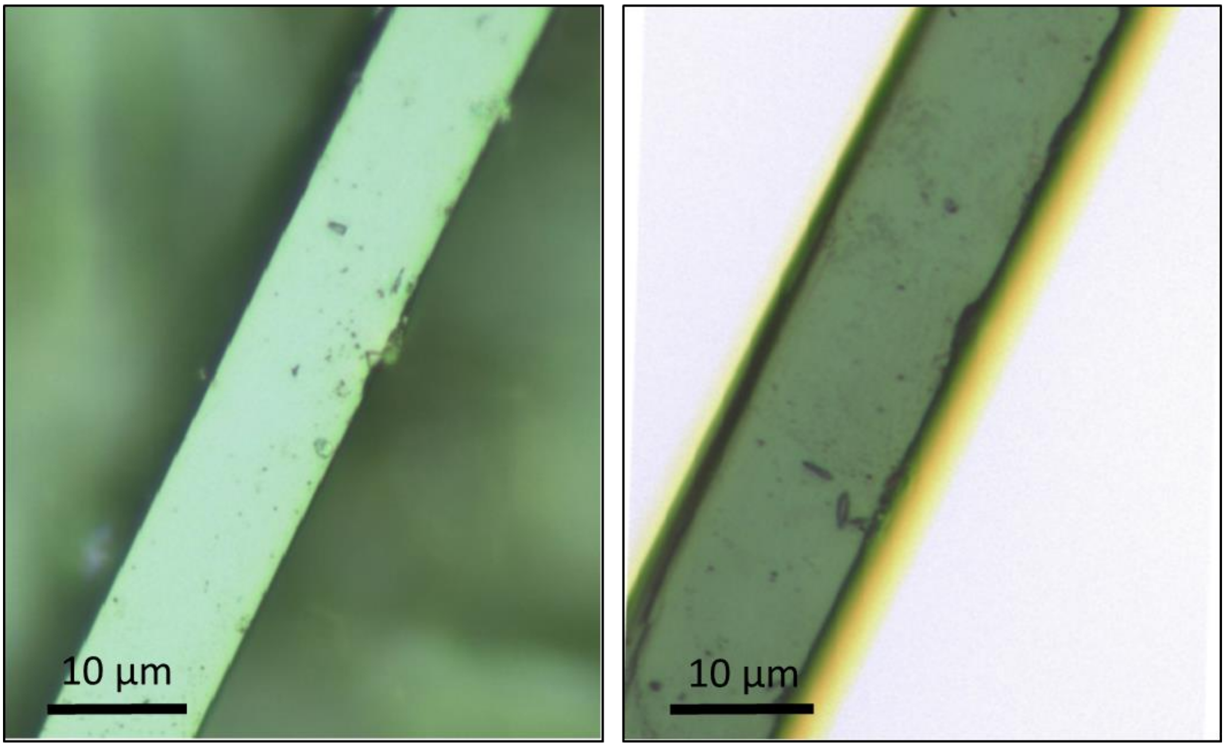

Supplementary Figure S1: MOF crystal optical images. Optical microscope images of the a Ce-RPF8 crystal, before (left) and after being immersed in pure water (pH=5.5) for 24 hours (right).

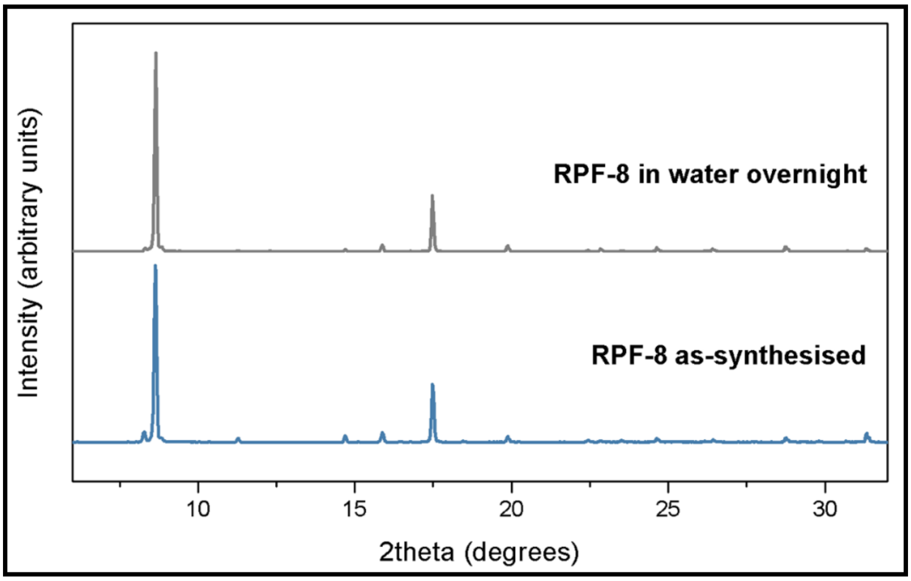

Supplementary Figure S2*: MOF crystal PXRD pattern.* Comparison of PXRD pattern for as synthesized RPF-8 crystals, before and after being immersed in water overnight.

Information about the supplementary videos

Supplementary Video S1. Imaged area = 235 nm x 235 nm in water. For the rest of the corresponding imaging parameters and processing see Fig. 2 specifications.

Supplementary Video S2. Imaged area = 50 nm x 50 nm in glycerol. For the rest of the corresponding imaging parameters and processing see Fig. 3 specifications. The last image of the video has an enlarged scan area of 80 nm x 80 nm and it clearly shows that no hole was produced by the imaging on the MOF surface.

Supplementary References

S1. www.gwyddion.net

S2. Guzman, H. V., Garcia, P. D. & Garcia, R. Dynamic force microscopy simulator (dForce): a tool for planning and understanding tapping and bimodal AFM experiments. *Beilstein J. Nanotechnol.* **6**, 369-379 (2015).
